# Exposure to anesthetic gases and Parkinson’s disease: a case report

**DOI:** 10.1186/1471-2377-13-194

**Published:** 2013-12-09

**Authors:** Giuseppe Mastrangelo, Vera Comiati, Massimiliano dell’Aquila, Emanuele Zamprogno

**Affiliations:** 1 Department of Molecular Medicine, Padua University, Padua, Italy; 2 Department of Anatomical, Histological, Medico-Legal and Locomotor Sciences, La Sapienza University, Rome, Italy

**Keywords:** Parkinson’s disease, Anesthetic gases, Nitrous oxide, Halothane, Isoflurane, Levoflurane, Occupational disease, Causality

## Abstract

**Background:**

The administration of anesthetics determines depression of the central nervous system and general anesthesia by inhalation may cause an environmental pollution of the operating rooms. It may therefore conceive a possible occupational etiology of Parkinson's Disease (PD).

**Case presentation:**

In a Caucasian male aged 59 years, PD was diagnosed by brain scans with a presynaptic radioactive tracer of the dopaminergic system. Family history was negative for Parkinson’s disease or essential tremor. He was a smoker, a moderate consumer of coffee and alcohol, and never exposed to pesticides/metals. For 30 years (since the age of 29 until today), he worked as an anesthesiologist in private clinics in the Veneto (Northern Italy), exposed to anesthetic gases. The time elapsed from first exposure to onset of disease is 22 years, fulfilling the requirement of the induction/latency period. A literature search demonstrated unacceptable levels of anesthetic gases in public hospitals of the Veneto region from 1990 to 1999. This exposure was presumably high also in private hospitals of the region until at least 2007, when an overexposure to sevoflurane was repeatedly measured in this patient. The association between occupational exposure to anesthetic gases and risk of Parkinson’s disease was supported by a case-control study (reporting a two-fold increase in the risk of PD associated with a clinical history of general anesthesia) and a cohort study comparing mortality from PD between US anesthesiologists and internists (showing a statistically significant excess (p=0.01) in anesthesiologists compared to internists). Numerous recent mechanistic studies (in vitro essays and in vivo short-term studies) strengthened the association between exposure to anesthetic gases (nitrous oxide, halothane, isoflurane, levoflurane) and PD.

**Conclusion:**

In view of the limited evidence of human studies and the sufficient evidence of experimental studies, the high exposure to anesthetic gases could have induced PD in the subject under study.

## Background

The main structures involved in the development of Parkinson’s Disease (PD) are the Substantia Nigra Pars Compacta and Striate Nucleus of the central nervous system (CNS), in which dopamine is the neuro-transmitter molecule.

The administration of anesthetics determines their distribution to the CNS and other tissues of the body. The desired effect is the depression of the CNS that is obtained in relation to the concentration achieved in this tissue. It is believed that general anesthesia by inhalation may cause an environmental pollution of the operating rooms and create problems of occupational exposure in operators. It may therefore conceive a possible occupational etiology of PD.

A case of Parkinson’s disease occurring in an anesthesiologist came recently to our attention. In order to evidence an association between occupational exposure to the agent and the disease, we made an extensive search of the literature. First, the patient was visited to confirm the diagnosis of PD and investigate the occupational exposure to volatile anesthetics.

## Case presentation

The patient (male, Caucasian, aged 59 years) worked for 30 years as a free lance anesthesiologist in surgical rooms of private clinics in the Veneto (North Eastern Italy), exposed to different anesthetic gases: nitrous oxide (N_2_O) for 22 years (until 2005); halothane, isoflurane, levoflurane and other halogenated aliphatic hydrocarbons for seven years (1983-1990); and sevoflurane for 23 years (from 1990 until now). In his post-shift urine, hexafluoroisopropanol (metabolite of sevoflurane) was 483.4 μg/liter (above the threshold of 465 μg/liter) in May 2007 and 535.6 μg/liter in a separate working day one week later.

The patient was born from a spontaneous delivery (his mother was 28) and always had regular habits. In particular, he reported the consumption of 5-6 cigarettes/day, half a liter/day of wine, 3 cups/day of coffee and tap water only. He always carried out sport (football, tennis, skiing). He denied environmental exposure to pesticides or heavy metals (iron, copper, manganese, lead) and disclaimed past health problems such as high blood pressure or vascular disorders, asthma, nor mood disorders, trauma or surgical interventions. He experienced a knee injury with lesion of cruciate ligaments (treated with ibuprofen/piroxicam and several arthrocentesis). In 2006, following recurrent left lumbosciatalgia he underwent nuclear magnetic resonance that demonstrated medium grade spondylolisthesis. Neurological symptoms of PD first appeared in 2005 and became progressively worse, leading to neurology consultations and imaging examinations. Brain magnetic resonance imaging was negative. The single-photon emission computed tomography (SPECT), with a radioactive presynaptic tracer of the dopaminergic system (123I DatSCAN), showed “reduced concentration of the tracer in the basal ganglia on the right and in the lenticular basal ganglia on the right and left, supporting the clinical suspicion of PD”. The subsequent treatment with dopaminergic drugs reduced symptoms.

### Exposure to anesthetic gases

**Figure 1:**
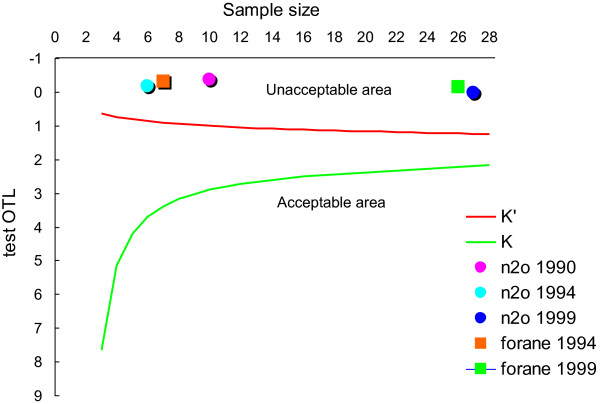
Compliance of exposures to anesthetic gases with exposure threshold values, assessed with the “one-sided tolerance limits” (OTL) test: OTL on y-axis and number of samples on x-axis of the OTL diagram.

The search of “occupational exposure”, “anesthetic gases” and “Veneto” in PubMed and Scopus returned three citations [1-3]. Available exposure data, collected with personal sampling in anesthesiologists in Veneto public hospitals from 1990 to 1999, are shown in Table 1: means and standard deviations as well as percentage of exceedance with respect to a threshold of 50 ppm for nitrous oxide and 2 ppm for forane. Exposure over time decreased for N_2_O but not for forane. Compliance with exposure threshold values was assessed with the “one-sided tolerance limits” (OTL) test, which places the statistic OTL (y-axis) against number of samples taken (x-axis) [4]. As shown in Figure 1, all measures were plotted in the unacceptable area.

**Table 1:** Anesthetist exposure to anesthetic gases (nitrous oxide and isoflurane) measured in the operating rooms of the Veneto Region, 1990-2002, by personal sampling: survey year, number of samples, mean and standard deviation (in parts per million, ppm), percentage of measurement exceeding the standard (Exceedance %), and references

**Year**	**N. Samples**	**Mean (ppm)**	**Standard deviation (ppm)**	**Exceedance %**	**References**
Nitrous oxide					
1990	10	259.25	108.42	100	[1]
1994	7	107.1*	135.5*	–	[3]
1999	27	46.9	55.5	33.3	[2]
Forane					
1994	7	2.88*	3.4*	–	[3]
1999	27	2.5	4.5	14.8	[2]

*environmental concentration values recalculated from the urine concentration.

### Epidemiological and experimental research

Different combinations of “anesthesiologist”, “anesthetic gases”, “nitrous oxide”, “halothane”, “isofluorane”, “sevofluorane”, “Parkinson’s disease”, “dopamine”, “dopaminergic neurons”, “substantia nigra”, “striatum”, “vitamin B_12_”, “cobalamine”, “αβ–amyloid” were searched in PubMed and Scopus.

An association between exposure to anesthetic gases and PD was suggested for the first time by a case–control study conducted in Italy (136 patients diagnosed with PD by a neurologist and 272 controls matched by age and sex), in which the risk of PD was evaluated with odds ratio (OR) and 95% confidence interval (CI) estimated with logistic regression analysis. Smoking adjusted ORs were: 41.7 (CI: 12.2–142.5; p <0.0001) for family history of PD; 10.8 (2.6–43.7; p<0.0001) for family history of essential tremors; 2.6 (1.4–3.7; p < 0.0013) for advanced maternal age at the time of childbirth; 7.7 (1.4–44.1; p<0.0212) for employment in agriculture; 2.0 (1.1–3.6; p < 0.0308) for the use of well water; and 2.2 (1.3–3.8; p<0.0024) for general anesthesia [5].

A cohort study compared mortality from PD between two large groups of US male doctors (33,040 anesthesiologists and 33,044 internists) that were followed up from 1979 to 1995 [6]. The standardized mortality ratio (SMR) was estimated separately in anesthesiologists and internists in two follow up periods (≤10 e >10 years) and, in each follow-up period, a risk ratio (RR) (ratio of SMR anesthesiologists / SMR internists) was obtained. Table 2 shows that in each group of doctors, mortality was lesser with respect to that in the US population. When the two groups were directly compared, RR becomes 3.47 (1.37–9.43) in the second period, a statistically significant excess (p = 0.01) indicating that PD risk was higher in anesthesiologists compared to internists [6].

**Table 2:** Mortality for Parkinsons disease as the underlying cause of death in two groups of specialized doctors (anesthetists e internists) from 1979 to 1995

**Length of follow up**	**Anesthetists**		**Internists**		**Comparison Anesthetists vs Internists**
	**SMR**	**CI**	**SMR**	**CI**	**RR**
All periods	1.33	0.75–21.5	1.03	0.61–1.59	1.29
≤ 10 years	0.60	0.15–1.57	1.78	0.89–3.12	0.34
> 10 years	2.22	1.15–3.80	0.64	0.27–1.24	3.47

The mechanistic studies linking exposure to anesthetic gases to PD are reported below.

According to Brodsky [7] nitrous oxide inactivates vitamin B_12_, which is the essential cofactor for the enzyme methionine synthetase. The latter favors the conversion of homocysteine (HC) in methionine, which is the precursor of S-adenosyl-methionine, a universal methyl-group donor involved in the regulation of DNA epigenetic processes [8]. The advent of sensitive diagnostic tests, including HC assays, has revealed a surprisingly high prevalence of a more subclinical form of B_12_ deficiency (without anemia), particularly in operating theatre staff exposed to high level of N_2_O [9]. Metabolic evidence of B_12_ deficiency was reported in association with several neurodegenerative disorders, including Parkinson’s disease [10]. Furthermore, in a murine model, deprivation of vitamin B_12_ produced apoptosis of neurons in Substantia Nigra of adult rats and appearance of a parkinsonian-like phenotype [11].

Isoflurane is metabolized by cytochrome P450 2E1 (CYP2E1); on the other hand CYP2E1 has been detected in Substantia Nigra Pars Compacta and Striate Nucleus. Animal studies and in vitro essays on neuronal tissues suggested that administration of isoflurane increased the production of reactive oxygen species (ROS), through enzyme induction of CYP2E1, implicated in the pathogenesis of PD [12].

Isoflurane anesthesia enhanced extracellular dopamine concentration through inhibition of dopamine active transporter (DAT), placed on dopaminergic pre-synapses [13,14]. The lesser recovery of dopamine from synaptic spaces involved an increase in: dopamine uptake, dopamine intracellular overload and ROS production at cytosolic level [15,16].

Isoflurane can induce neuro-inflammation, as shown by different studies that found an increase of tumor necrosis factor (TNF–alfa) and interleukins (IL–6 e IL–1beta) in the cerebral tissues of rodents [17].

Isoflurane reduced (by facilitating endocytosis) synaptic receptors n–methyl–d–aspartate [NMDA] in an experiment in vitro where neurons from healthy mice were exposed to isoflurane 2% for six hours. There were ensuing changes in excitability of nigrostriatal and corticostriatal neurons that greatly reduced cognitive performance and played a role in the onset of PD [18].

Sevoflurane was found to increase TNF-alfa and production of beta-amyloid through activation of caspases and apoptotic cascade [19], which are major characteristics of PD [20].

Halothane and isoflurane increased cytotoxicity in pheochromocytoma cells in vitro, by increasing oligomerization of beta-amyloid. The same mechanism presents itself at the nigro-striate level producing dopaminergic neurotoxicity [21].

Halothane has been shown to be capable of binding tubulin, altering its polymerization and destroying the polymerized microtubules [22]; in addition, a recent study has shown an interaction between the halothane molecule and the microtubular elements of the neuronal cytoskeleton [23]. High dose of anesthetics cause an alteration in the structure of tubulin, which in turn impedes the polymerization of the microtubules, thus determining a lack in macrotubule formation [24]. This evidence suggests a role of anesthetics in the exacerbation of neurodegenerative illnesses such as PD [22].

## Conclusion

The diagnosis of PD in this patient is quite certain since it is based on a brain SPECT demonstrating a reduced concentration of a presynaptic radioactive tracer of the dopaminergic system in the basal ganglia on the right and in the lenticular basal ganglia on the right and left.

Pollution from anesthetic gases at unacceptable levels was widespread in public hospitals in the Veneto from 1990 to 1999 (Table 1 and Figure 1) as well as in private hospitals in the Veneto until at least 2007, as it can be inferred by overexposure to sevoflurane assessed in the patient while working in one of these private structures. The time elapsed from first exposure to anesthetic gases (1983) to onset of PD symptoms (2005) was 22 years, fulfilling the induction/latency period of >10 years that was observed in the US study comparing anesthesiologists and internists [6].

The hypothesis of association between exposure to anesthetic gases and PD is recent [5,6] and PD is relatively rare; both facts explain the few number of epidemiological studies on the topic. However, the hypothesis of association between exposure to anesthetic gases and risk of PD was strengthened by several recent mechanistic studies (in vivo short-term studies and in vitro essays) that linked exposure to anesthetic gases (nitrous oxide, halothane, isoflurane, lovoflurane) with PD risk.

In the reported case, moreover, a non-occupational disease is unlikely because of the absence of: family history for PD or essential tremor and personal history of mood disorders [25]; bronchial asthma constipation and arterial hypertension (or treatment with beta blockers or calcium channel blockers) [26]; general anesthesia [5]. There was no exposure to pesticides, residency in rural areas or use of well water contaminated by pesticides [5,15]. The patient is a smoker and a moderate consumer of coffee and alcohol though these habits are protective factors against PD [25].

In view of the limited evidence of human studies and the sufficient evidence of experimental studies, the high exposure to anesthetic gases could have induced PD in the subject under study. Although additional studies are warranted, this evidence could enable Health Authorities to appropriately answer the claims of workers exposed to anesthetic gases, taking into account scientific evidence and social expectations.

## Consent statement

Written informed consent was obtained from the patient for publication of this case report. A copy of the written consent is available for review by the Editor-in-Chief of this journal.

## Competing interests

Authors declare that they do not have any financial and non-financial competing interests, such as:

● receiving any reimbursements, fees, funding or salary in the past five years from an organization that may in any way gain or lose financially from the publication of this manuscript;

● holding any stocks or shares in an organization that may in any way gain or lose financially from the publication of this manuscript;

● currently applying for any patents relating to the content of the manuscript;

● having political, personal, religious, ideological, academic, intellectual, commercial or any other in relation to this manuscript.

## Authors’ contributions

GM conceived the study, participated in its design and coordination, and helped to draft the manuscript. VC searched the literature and helped to draft the manuscript. MdA critically revised the manuscript for important intellectual content. EZ participated in the design of the study, helped to draft the manuscript, and performed the statistical analysis. All authors read and approved the final manuscript.

## Authors’ information

GM is Associate Professor in Occupational Medicine, Responsible of Laboratory of Occupational Epidemiology at Department of Molecular Medicine, University of Padova (Italy); author of 172 papers in peer-reviewed journals (1655 citations; H index = 24; overall IF = 294.061); associate editor and reviewer for many scientific journals. VC is a post-graduate student in Occupational Medicine at University of Padova (Italy). MdA is a post-graduate student in Forensic Medicine at La Sapienza University, Rome (Italy). EZ is a post-graduate student in Occupational Medicine at University of Padova (Italy).

## Pre-publication history

The pre-publication history for this paper can be accessed here:

http://www.biomedcentral.com/1471-2377/13/194/prepub
